# Modelling the Relative Abundance of Roe Deer (*Capreolus capreolus* L.) along a Climate and Land-Use Gradient

**DOI:** 10.3390/ani12030222

**Published:** 2022-01-18

**Authors:** Caryl S. Benjamin, Lars Uphus, Marvin Lüpke, Sandra Rojas-Botero, Maninder Singh Dhillon, Jana Englmeier, Ute Fricke, Cristina Ganuza, Maria Haensel, Sarah Redlich, Rebekka Riebl, Cynthia Tobisch, Johannes Uhler, Jie Zhang, Annette Menzel, Wibke Peters

**Affiliations:** 1 Ecoclimatology, TUM School of Life Sciences, Technical University of Munich, 85354 Freising, Germany; caryl.benjamin@tum.de (C.S.B.); lars.uphus@tum.de (L.U.); luepke@wzw.tum.de (M.L.); 2 TUM School of Life Sciences, Chair of Restoration Ecology, Technical University of Munich, 85354 Freising, Germany; sandra.rojas-botero@tum.de; 3 Institute of Geography and Geology, Department of Remote Sensing, Julius-Maximilians-University Würzburg, 97070 Würzburg, Germany; maninder.dhillon@uni-wuerzburg.de; 4 Field Station Fabrikschleichach, Department of Animal Ecology and Tropical Biology, Julius-Maximilians-University Würzburg, 97070 Würzburg, Germany; jana.englmeier@uni-wuerzburg.de (J.E.); johannes.uhler@uni-wuerzburg.de (J.U.); 5 Department of Animal Ecology and Tropical Biology, Julius-Maximilians-University Würzburg, 97070 Würzburg, Germany; ute.fricke@uni-wuerzburg.de (U.F.); cristina.ganuza_vallejo@uni-wuerzburg.de (C.G.); sarah.redlich@uni-wuerzburg.de (S.R.); jie.zhang@uni-wuerzburg.de (J.Z.); 6 Bayreuth Center of Ecology and Environmental Research (BayCEER), University of Bayreuth, 95440 Bayreuth, Germany; maria.haensel@uni-bayreuth.de (M.H.); rebekka.riebl@uni-bayreuth.de (R.R.); 7 Institute of Ecology and Landscape, Weihenstephan-Triesdorf University of Applied Sciences, 85354 Freising, Germany; cynthia.tobisch@hswt.de; 8 Bavarian State Institute of Forestry (LWF), Hans-Carl-von-Carlowitz-Platz 1, 85354 Freising, Germany; 9 Wildlife Biology and Management Unit, Technical University of Munich, 85354 Freising, Germany

**Keywords:** density surface model, distance sampling, roe deer, GAM, climate change, land-use, spatial modelling, extrapolation, dung pellets

## Abstract

**Simple Summary:**

The European roe deer is the most abundant ungulate in Europe. Information on the number of animals and the factors that influence this are essential for the effective management of this species. We developed a model to estimate the pellet group density using transect surveys of dung pellets in the federal state of Bavaria, south-eastern Germany. We used the pellet group density as a proxy for roe deer relative abundance. Our results show that climate, habitat type and wildlife management approach determine the relative number of roe deer. Influential factors differed between seasons and were expected due to changes in food and shelter availability. Although recognized as a woodland species, the majority of roe deer are expected in agriculture-dominated landscapes, which shows their ability to adapt to a wide range of landscape types, especially those with high food availability. Higher numbers were also predicted in regions with intermediate temperatures. Estimates of relative number do not give the actual absolute number of animals but are useful in determining which conditions will have more or fewer animals and can provide information for broad-scale management recommendations. Our results also provide insights into possible future changes in the distribution of relative numbers of roe deer due to climate and land-use change.

**Abstract:**

European roe deer (*Capreolus capreolus* L.) are important given their economic, recreational and ecological value. However, uncontrolled roe deer numbers can result in negative impacts on forest regeneration and agricultural crops, disease transmission and occurrences of deer-vehicle collisions. Information on the abundance and distribution is needed for effective management. We combined distance sampling (DS) of roe deer dung pellet groups with multiple variables to develop a density surface model (DSM) in the federal state of Bavaria in south-eastern Germany. We used the estimates of pellet group density as a proxy for roe deer relative abundance. We extrapolated our best DSM, conducted a quantitative evaluation and contrasted relative abundance along climate and land-use gradients. Relative abundance of roe deer was influenced by a combination of habitat type, climate and wildlife management variables, which differed between seasons and which reflected changes in food and shelter availability. At the landscape scale, the highest abundance was observed in agriculture-dominated areas and the lowest in urban areas. Higher abundance was also observed in areas with intermediate temperatures compared to the warmest areas. Our results provide information on possible future changes in the distribution of relative abundance due to changes in climate and land-use.

## 1. Introduction

European roe deer (*Capreolus capreolus* L.) populations have increased dramatically in numbers and geographical distribution in recent decades [1]. This is indicative of the species’ ability to adapt to a wide range of landscape types and climatic conditions [2,3]. Effective management of this species is important because of its economic (meat and hunting), recreational (hunting, wildlife photography) and ecological value (biodiversity) [4]. However, the current trend of increasing population sizes in multi-use landscapes has led to numerous conflicts such as damage to agricultural crops, compromised forest regeneration due to selective browsing, disease transmission and an increase in collisions between deer and vehicles [5,6,7,8,9]. Therefore, effective future wildlife management strategies, which require information on abundance and distribution, are needed [10].

Wildlife monitoring include direct and indirect methods. Direct methods involve counting individuals of the target species, while indirect methods focus on counting traces left by animals (e.g., nests, tracks, dung), which can be converted to the absolute number of animals using multipliers [11,12,13]. There have been numerous attempts to estimate ungulate population sizes using various methods, including direct line transect surveys [14,15], thermal imaging [16,17] or camera surveys [18,19] and indirect pellet group counts [20] (methods reviewed in [21], population density estimates in [22]). However, the ability to estimate abundance gradients on a larger geographical scale is limited by the high monetary and labor costs of these methods [12]. Because of these limitations, Morellet et al. [23] argued for a shift in focus from estimating absolute abundance to understanding the interaction between animal populations and habitat quality and determining the ecological status of the ungulate-habitat system. They suggested that relative abundance indices might be sufficient for management decisions.

Previous studies have highlighted the importance of shelter or the combination of food and shelter for roe deer [22,24,25]. Natural predators were identified as important top-down control in some populations [22]. Especially in the absence of large predators, hunting is often considered an important management tool for population control of roe deer [26,27]. Climate-related variables, such as severe winter conditions, spring temperatures and precipitation that affect forage quality, and droughts in summer, can also contribute to population dynamics [28,29,30].

Given the importance of habitat and climate-related variables for roe deer density and the current trend of human-induced landscape and climate changes, we estimated the relative abundance of roe deer along a climate and land-use gradient. We used an indirect method of dung-pellet-based distance sampling because it accounts for imperfect detection and has been shown to be a robust method for estimating deer abundance [11,20]. Density surface modelling allowed us to estimate relative abundance at a larger scale, including areas not covered by the field survey [31]. This method has also been used to estimate the abundance of roe deer in the Mediterranean, but at a smaller spatial scale [32]. Other examples of method application include estimation of abundance and distribution of various species, such as birds [33], marine [34] and terrestrial mammals [35]. We then utilized a recently developed workflow to evaluate extrapolation quality [36].

Specifically, we aimed to (1) model the seasonal relative abundance of roe deer (2) determine the relationship between relative abundance and various habitat, climate and management variables, (3) quantitatively evaluate the spatial extrapolation, (4) compare relative abundances at sites within a climate and land-use gradient and (5) assess the feasibility of the methodology for state-wide data-driven management decisions. We expected the relative abundance of roe deer to be affected by a combination of climate, land-use and wildlife management variables. Lowest abundances were expected both in the coolest climate zone, due to harsher winters, and in the warmest one, due to higher risk of drought in summers. In addition, we expected that densities in land-use types would vary seasonally due to changes in food and shelter availability in the forests and open areas.

## 2. Materials and Methods

### 2.1. Survey Region

**Figure 1:**
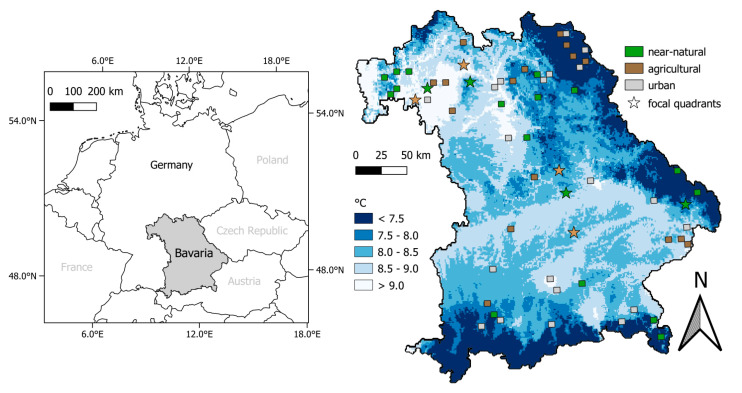
Distribution of the study regions in Bavaria along a climate and land-use gradient. Each box/star represents one of the 60 quadrants with color showing the dominant land-use. Higher sampling intensity was done in the focal quadrants (☆). Background color represents mean annual temperature (1981–2010, [39]).

This study was conducted in the federal state of Bavaria, south-eastern Germany (Figure 1), which is the largest federal state, with an area of 70,000 km^2^ [37]. It is characterized by diverse climates and high heterogeneity of habitats. The area is composed of 53% agricultural and 7% urban; the remaining areas are predominantly managed forests, nature protection regions, and near-natural habitats (40%, [38]).

Sixty study regions were selected based on an existing 5.8 km × 5.8 km grid (hereafter, quadrants) covering the whole of Bavaria (‘TK25′ topographical map established in 1868, scale 1:25,000; Figure 1). These quadrants were divided into five climatic zones (climate 1: <7.5 °C, climate 2: 7.5–8 °C, climate 3: 8–8.5 °C, climate 4: 8.5–9 °C and climate 5: >9 °C, based on multi-year mean temperature 1981–2010, [39]) and three landscape types based on the dominant land-use (near-natural, agricultural and urban). Land-use was classified using CORINE (2012) land cover classification with the following classes: quadrants classified as ‘near-natural’ consist of more than 85% near-natural vegetation, with a minimum of 50% forest; ‘agricultural’ quadrants of more than 40% arable land and managed grassland; lastly, ‘urban’ quadrants contain more than 14% housing, industry and traffic infrastructure. Based on these classifications, 4 replicate quadrants were selected for each of the 15 (5 times 3) climate-land-use classifications, yielding a total of 60 quadrants.

Using a novel correlation heatmap approach [40], three local study plots (0.5 ha) with different local land-use types (forest, grassland, arable land, or settlement) were identified in each quadrant as sites for different research initiatives related to biodiversity and ecosystem services, including this study. The heatmaps were created for each local land-use type in each quadrant to visualize the correlation between landscape composition (proportional cover of each land-use type) and configuration (edge density) in the entire quadrant. Local study plots were established in sites with the lowest possible correlation between landscape composition and configuration. The selection of quadrants and study plots is described in detail in Redlich et al. [40].

### 2.2. Field Survey

We combined two types of distance sampling surveys with different sampling intensities for roe deer dung pellet groups using 200-m line transects in spring-summer and autumn of 2019 [31,41]. First, a designed survey, where transects were randomly placed across the entire quadrant, was applied in eight so-called “focal” quadrants, covering the climate classes 1–4 and landscape types near-natural and agricultural (Figure 1). Here, surveys were designed using the software Distance 7.3 [13]. The design class was a systematic segmented trackline with 75% effort in woodlands and 25% in open areas (excluding settlements), which translated to 16 to 29 transects per quadrant. Stratification of the design based on habitat-type was done using the CORINE [42] land-use classification map. The survey design was originally planned for 9 focal quadrants and generated 206 transects. We later decided to do the surveys in eight quadrants for a balanced design (four near-natural quadrants, four agricultural quadrants). Out of 206 transects generated by the software, 150 transects were sampled in the focal quadrants in spring and 168 transects in autumn. In addition to the transects from the discarded quadrant (*n* = 24), more transects were skipped because these were too difficult to access due to steepness, very dense vegetation or being located in arable land with crops. The different land-uses were, however, still properly represented, with a 75:25 woodland to open area ratio in the number of transects in spring and 77:23 ratio in autumn. In many cases, the survey was still done in arable land with crops, but at the border. All transects in the designed survey had a west–east direction.

For the second type, undesigned distance sampling surveys were conducted in both focal and non-focal quadrants across Bavaria. One to two 200-m transects were sampled in 52 (out of 60) quadrants in spring and 56 quadrants in autumn, with one transect in the forest study plot and another in the arable or grassland study plot. The starting point of each transect was chosen randomly within a 30-m radius to the center of the study plot. The direction was towards a wildlife camera (data for another study) installed in areas with signs of ungulate presence (e.g., animal tracks, dung, etc.). The data for the spring model came from surveys done primarily in Spring (April and May, 83% of the transects) and were carried out by 26 observers from 2 April to 11 August 2019, while the autumn surveys were carried out by 12 observers from the 20 September to 11 November.

A roe deer dung pellet group was considered a detection if it contained at least five pellets that could fit inside the palm of the hand of the observer. Each detection was recorded as the perpendicular distance between the center of the dung pellet group and the transect line. Transitions in the ground cover within the transect were noted because this affects detectability; they were also used as a basis for segmenting the transects for the density surface model. Ground cover classes were: bare ground, grass and forb, shrub with height less than 30 cm, height 30 to 100 cm, and height of more than 100 cm.

### 2.3. Model Development

We used the two-stage approach proposed by Hedley and Buckland [43], wherein the detection function is modelled in the first stage and density surface modelling (DSM) in the second stage. The detection function is a model of the probability of detecting a dung pellet group at a distance *x* from the transect line, wherein *x* is equal to or more than 0 and less than or equal to the truncation distance (0 ≤ *x* ≥ truncation distance). Next, the DSM approach was used to predict spatially explicit pellet group density as a function of significant variables using a generalized additive model (GAM, [44]). The DSM model outcome was pellet group density; however, this was used as a proxy for roe deer relative abundance. The two terminologies are, therefore, equivalent in this study. These two stages were implemented in R 4.0.2 [45] using the packages distance [46] and dsm [31].

Because this study aimed to estimate relative abundance in spring and autumn to account for seasonal differences in food and shelter availability, separate detection functions were fitted for spring and autumn data. All distance data were truncated at 1.5 m, discarding approximately the farthest 5% of the data [13]. We assessed the effect of observation-level covariates on the detection probability using multiple covariate distance sampling (MCDS) [47]. Detection probability is the probability of detecting a dung pellet group given its distance from the transect and as a function of a set of covariates. Covariates tested were the observer group and the type and height of the ground cover. Observers were post-stratified according to the total length (effort) of transect survey that they conducted into groups of a minimum of 70 detections in total, which is a prerequisite for fitting detection functions [41]. This led to five observer groups in spring-summer and three groups in autumn. Detections were fitted with half-normal and hazard-rate key functions with (cosine, hermite, simple polynomial) and without adjustment terms [48]. Models with covariates did not include adjustment terms. The best model was selected using Akaike’s information criterion (AIC). Model evaluation was done using the Cramér–von Mises goodness of fit criterion [13].

We started the second stage in model development (DSM) by segmenting our transects based on habitat transition points identified during the field surveys. Segment lengths ranged from 3 m in heterogeneous to 200 m at homogenous survey sites. For each segment centroid, a buffer with a radius of 500 m was generated, representing the average roe deer home range size (if simplified as a circle) in Germany at a monthly scale [49]. These buffered segment centroids were characterized by a combination of habitat-related features (land-use [42], topography [50], edge proximity from CORINE forest class [42], NDVI [51]), climate variables [52,53,54,55], and forest ownership type [56] as a proxy for management differences at different resolutions ranging from 25 m to 1000 m (Table 1). We reclassified the land-use classes in CORINE [41], as shown in Table S1. Climate variables (mean temperature, temperature range and precipitation) were retrieved from the seasonal gridded product of the German Meteorological Service [52,53,54,55] for the year 2019. Spring, summer, autumn, and winter refer to the standard meteorological seasons (MAM, JJA, SON, DJF, respectively). The seasonal temperature range was calculated as the difference between the monthly averaged daily maximum and minimum air temperature [53,54]. Covariates were summarized as the mean of all values within these buffered regions or as percentage cover for the land-use and forest ownership variables.

A Horvitz–Thompson-like estimator was applied to estimate abundances in each segment; this was modeled with a tweedie or negative binomial distribution as a function of the sum of the smooth functions of different habitat, climate and management related covariates. The covariates were first tested for collinearity and one of two correlated covariates (Pearson’s r > |0.7|) was discarded based on ecological significance (e.g., elevation discarded instead of winter mean temperature and summer temperature range). Thin plate regression splines (TPRS) [44] were used to automatically select knot positions. Model selection was done using a backward stepwise approach until all approximate p-values were significant (*p* < 0.05) [57]. There were, thus, four candidate models, two (tweedie and negative binomial distribution) for spring and two for autumn [31]. The best model for each season was selected by comparing several established metrics: AIC, percent deviance explained and comparison of quantile-quantile plots [44]. 

### 2.4. Prediction and Uncertainty Analysis

Using the spring and autumn DSM, we predicted the abundance of dung pellet groups for the whole of Bavaria on a 1 km × 1 km grid. We used this estimated density (n/km^2^) as a measure of relative abundance. We did not convert the abundance of dung pellet groups to animal abundance, as this would have required region-specific estimates of production and decay rates, which were not available. We used the delta method to estimate the uncertainty associated with the abundance estimate in each 1-km^2^ cell [58].

The coverage of the field survey was very limited relative to the prediction area, so we conducted a quantitative and spatially explicit evaluation of the extrapolation [59,60] using the R package *dsmextra* [36]. We used the two proposed metrics: the extrapolation detection (ExDet) tool [59] and the percentage of data nearby (%N) [61,62]. The extrapolation detection tool computes an index that can be classified according to three classes: (1) analogue, wherein the prediction was interpolated in a particular grid cell, (2) combinatorial, wherein the novel combinations of values encountered are within the univariate range of reference covariates and (3) univariate or when the prediction was made using values that were outside the range of each covariate in the DSM. The percentage of data nearby (%N) for any grid cell was quantified as the proportion of survey data within a given radius that was used as the basis for prediction [60]. The value of the radius is the mean Gower’s distance between all pairs of reference points. The theory behind this metric is that the reliability of predictions depends on the density of nearby observational data [36,62]. Based on the extrapolation evaluation metrics results, extrapolated (univariate) predictions with a %N of less than 5% were discarded.

There are no abundance estimates for roe deer for all of Bavaria. As an alternative to validation with an independent abundance dataset, we tested for a possible relationship between our estimated relative abundance and the total number of roe deer killed by hunting (hereafter, harvest) for 2019 using non-parametric Spearman correlation. Harvest data from official government statistics were expressed as densities for each game management district (n/km^2^) and estimated pellet group densities (n/km^2^) were averaged for all grid cells within a game management district. A game management district was excluded from the comparison if the total area of valid predicted cells (after extrapolation evaluation) within the district was less than 50% of the hunting district area. Out of the 746 game management districts in Bavaria, 506 were included in the autumn comparison (240 excluded) and 322 in the spring comparison (424 excluded).

### 2.5. Relative Abundance in a Climate and Land Use Gradient

We then presented the estimated distribution of the relative abundance of roe deer within the 60 study quadrants, which were classified according to the climate (class 1–5) and land-use (near-natural, agricultural and urban) gradients. Separate boxplots were made for harvest data as well as spring and autumn abundance predictions to compare trends along the gradients visually. These were not statistically compared because values are model predictions and therefore have smoothed variability which does not satisfy independence.

## 3. Results

A total of 1680 dung pellet groups were recorded on 512 transects of 200-m length, corresponding to a total effort of 102.4 km. After truncation, 891 detections were used to develop the habitat-based density model in spring, while 700 detections were used for the autumn model. The average number of detections per 200-m transect was 6.1 (standard deviation (sd) 8.10) in spring and 4.8 (sd 5.15) in autumn, and the maximum number of detections was 46 in spring and 24 in autumn.

### 3.1. Detection Function

**Figure 2:**
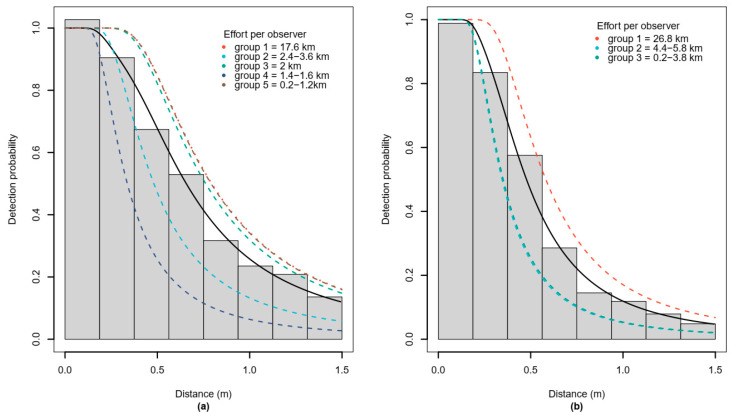
Distribution of observed distances between centers of roe deer dung pellet groups and the transect line: (**a**) spring, (**b**) autumn. Dashed lines show the probability of detection for each observer group. Observers were grouped according to the total length of transects they surveyed as a proxy for experience. The solid line is the average estimated detection function.

The best models for the detection function fitted using MCDS were those with a hazard rate key function without adjustment terms and observer group as covariate (Figure 2). Ground cover type and height had no significant effect on detection probability according to AIC model selection. Table S2 shows the AIC comparison results for all model versions for both spring and autumn detection functions. The average detection probability for spring was 0.50 with a coefficient of variation (CV) of 0.042. The average detection probability in autumn was 0.38 with a CV of 0.043. The selected detection function models also met the weighted Cramér-von Mises goodness-of-fit tests with *p* = 0.39 for spring and *p* = 0.49 for autumn.

### 3.2. Density Surface Models

Density surface models with a tweedie distribution were selected for both spring and autumn as these provided a better fit than the negative binomial in terms of AIC and percentage deviance explained (Table 2). Comparison of the quantile-quantile plots (Figure S1) also supports this choice.

The estimated abundance of roe deer pellets was in both seasons best explained by a combination of climate, habitat-related and management covariates (Table 2). Significant predictors in spring included both the mean and temperature range in winter, temperature range and accumulated precipitation in spring, the percent coverage of all habitat types (except artificial and water and other unsuitable areas, Table 1) and the percent coverage of privately-owned forests. Of all the climate-related variables, only the temperature ranges were significant in the autumn model. Moreover, edge proximity, the proportion of grasslands and shrublands, private and corporate forests were significant in the autumn model. 

**Figure 3:**
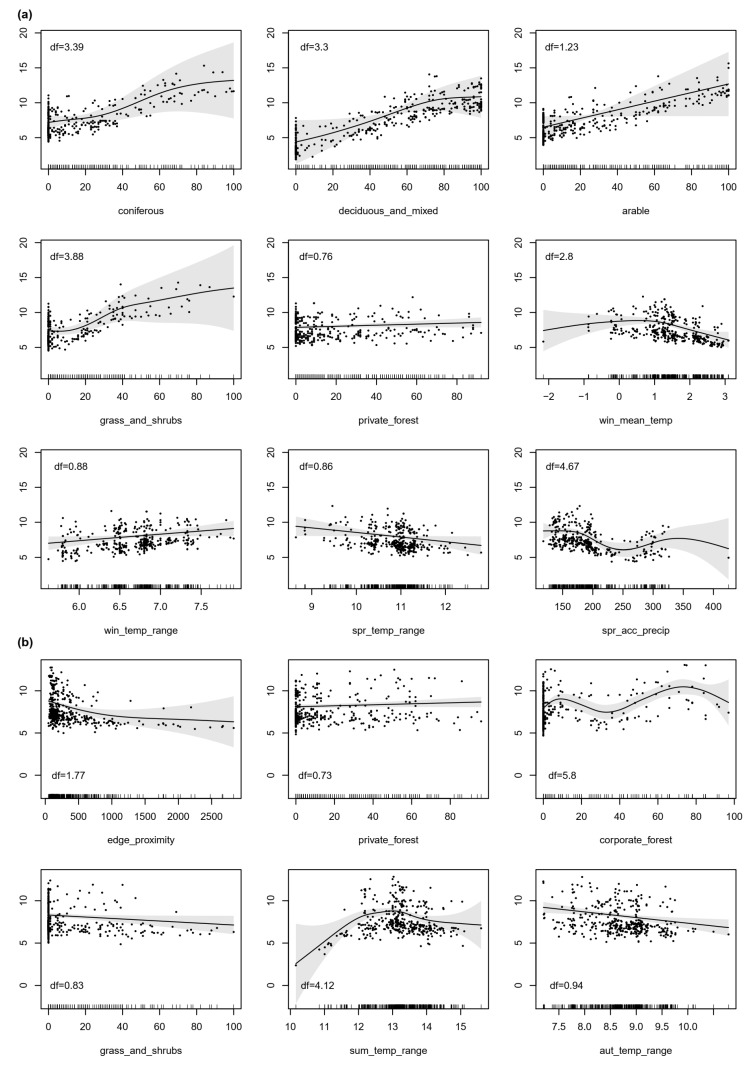
Partial effect plots of the estimated smooth functions (solid lines) for the selected covariates in the best models for (**a**) spring, and (**b**) autumn. Points are partial residuals. The scale of the *y*-axis is shifted by the value of the intercept so that each plot shows the prediction of dung pellet group abundance using the selected predictor and assuming that all other variables are kept at their average value. Degrees of freedom are shown in the upper left corner of the plots. Shaded regions correspond to 95% confidence intervals (CI). Some CIs are wider due to limited sampling coverage and effects are weaker in these areas. The one-dimensional scatterplot at the bottom of each graph shows the distribution of the available data. For variable names, see Table 1.

Areas with a higher proportion of forest, arable land, grass or shrubland cover were favorable for roe deer in the spring model (Figure 3a). Relative abundance was also predicted to be higher in areas with larger proportions of privately owned forests, with an intermediate winter mean temperature, a broader winter seasonal temperature range, a lower temperature range in spring, and medium cumulative spring precipitation. The range of the estimated effect of the selected predictors suggests that the habitat-related variables have a stronger influence on the estimated abundance than the climate and management variables.

Areas near forest edges were predicted to have a higher relative abundance of roe deer in the autumn model (Figure 3b). The percentage of private and corporate owned forests also had a significant positive effect on the abundance of roe deer pellet abundance. Fewer pellets were predicted in open habitat such as grasslands and shrublands. In terms of temperature, higher pellet abundance was predicted for medium temperature range in summer and a minimum range in autumn. Based on the estimated effects, areas with 70–80% of corporate forests contributed to higher pellet abundance. 

### 3.3. Prediction, Uncertainty and Validation

**Figure 4:**
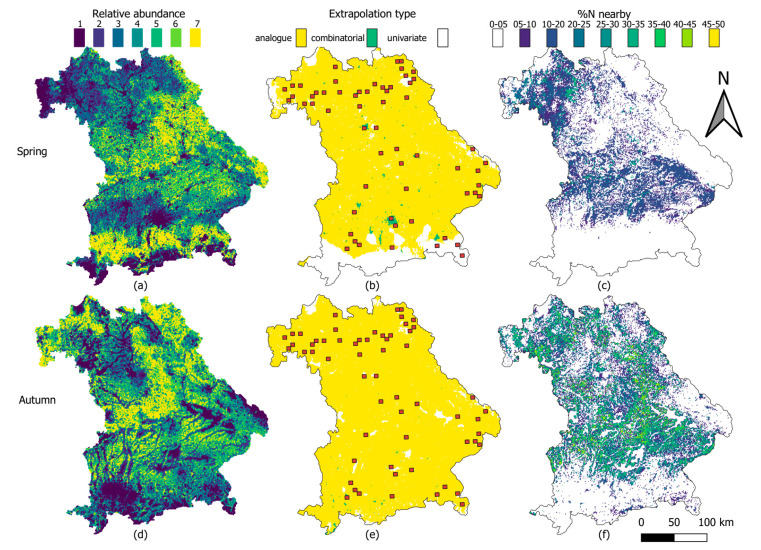
Prediction of relative roe deer pellet abundance and extrapolation evaluation of the density surface models in spring (**a**–**c**) and autumn (**d**–**f**). Relative abundance (**a**,**d**), extrapolation type (**b**,**e**) and calculation of the neighborhood metric of extrapolation (%N—**c**,**f**). Predicted relative abundance was divided into seven quantiles with 1 representing the lowest abundance and 7 the highest. The red squares in the extrapolation type maps (**b**,**e**) are the study quadrants where relative abundance was compared in a climate and land-use gradient.

The spring model predicted an interquartile range (IQR) of 668 to 4693 pellet groups with a mean of 3800 for an area of 1 km^2^, while the autumn model predicted an IQR of 827 to 5272 pellet groups with a mean of 4284. The spatial variability of the predictions of relative abundance is shown in Figure 4a for spring and Figure 4d for autumn.

Uncertainty associated with predicted abundance of dung pellet groups is provided by the CV around the abundance estimate. The CV from the detection function in the spring model was 0.04, while the CV of the GAM was 0.25. The total CV calculated using the delta method was 0.26. The autumn model also had a CV of 0.04 from the detection function and a lower CV of the GAM of 0.16. The total CV of 0.12 was consequently lower than for the spring model. Spatially explicit estimates of CV for both models are included in the supplementary material (Figure S2).

Quantitative and spatially explicit evaluation of the extrapolation showed that 13.06% (9026 grid cells) of the predictions in spring and 3.23% (2230 grid cells) in autumn were univariate (Figure 4b,e). Only a minority were combinatorial with 0.91% or 630 grid cells in spring and 0.13% or 88 grid cells in autumn. Analogue conditions (all values at the prediction site observed at the survey sites) were found for the majority of the grid cells in spring (86%, Figure 4b) and autumn (97%, Figure 4e). Of all the significant predictors in the spring model, the winter temperature range was the most influential covariate in univariate extrapolation. For the autumn model, edge proximity was the most influential covariate to univariate extrapolation.

Figure 4c,f show the quantitative measure of the amount of data available and used to support the prediction in each grid cell. Given the results of the extrapolation evaluation, model validation and further analysis were limited to areas that were analogue or combinatorial and with %N of more than 5%.

We found that there was moderate, but significant positive correlation of annual harvest densities with the spring model predictions (Spearman rank correlation r = 0.349, *p* < 0.01) and a lower, but also significant and positive, correlation of harvest densities with the autumn model predictions (Spearman rank correlation r = 0.136, *p* < 0.01).

### 3.4. Relative Abundance in a Climate and Land-Use Gradient

**Figure 5:**
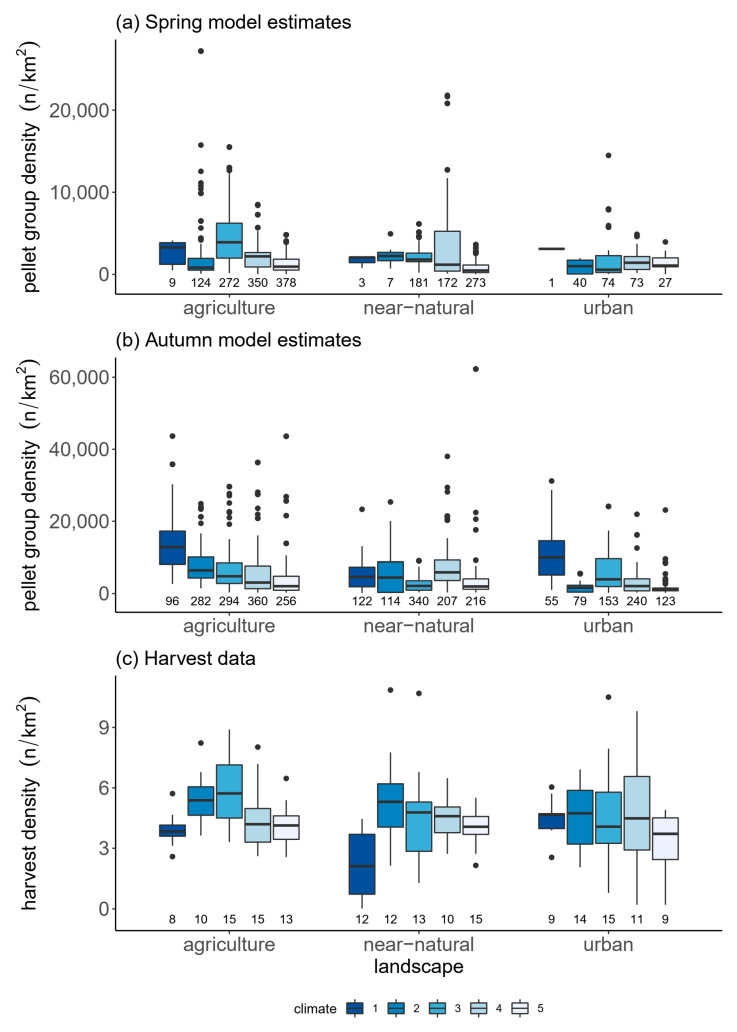
Relative abundance of roe deer in a climate and land-use gradient across Bavaria (**a**) spring model estimates, (**b**) autumn model estimates, (**c**) harvest data. Climate 1 to 5 indicates cooler to warmer mean multi-annual temperatures, which are inversely related to annual precipitation sums. The numbers at the bottom of each boxplot indicate the number of data points: number of 1 km^2^ grid cells for the spring (**a**) and autumn (**b**) models and number of game management districts for the harvest data (**c**).

The predicted relative abundance of roe deer in the different climate zones and land-use types in spring and autumn as well as in harvest data are presented in Figure 5. Harvest density was lowest in the coldest climate in both agricultural and near-natural landscapes. In contrast, relative abundance in the autumn model was highest at the coldest sites in the agricultural landscape and second highest in the near-natural landscapes. For the spring model, however, data was limited as only nine grid cells in the agricultural landscape and three in the near-natural landscape fell into this category. 

There was a general pattern of decrease in roe deer harvests from areas with intermediate temperatures (climate 3) to the warmest areas (climate 5). Harvest density in climate 3 in agricultural landscapes had a median value of 5.7/km^2^ as compared to 4.1/km^2^ in areas with climate 5. The same pattern was observed for near-natural landscapes with a median harvest density of 4.7/km^2^ in climate 3 and 4.1/km^2^ in climate 5. This pattern was equally predicted by both the spring and autumn models. In the spring model, the median pellet group density was 3909.5/km^2^ in climate 3 in agricultural landscapes and 942.2/km^2^ in climate 5. In the near-natural landscapes, it was 1805.8/km^2^ in climate 3 and 473.1/km^2^ in climate 5. In the autumn model, the median density in climate 3 in agricultural landscapes was 4798.1/km^2^ and 2041.4/km^2^ in climate 5. The only exception were the near-natural landscapes in the autumn model, where the median density in climate 3 (2104.9/km^2^) was almost the same as in climate 5 (1961.3/km^2^).

Relative abundance also differed between landscape types in both spring and autumn models with pooled data from the different climate classes. The highest predicted relative abundance of roe deer pellets was found in agricultural landscapes and the lowest in urban landscapes. Most importantly, the autumn model estimated higher relative abundance of roe deer at the study sites than the spring model (Figure S3).

## 4. Discussion

Conventional distance sampling has been used to estimate abundance of ungulates [63,64] including roe deer [65,66]. Here, we used the model-based approach of density surface modelling (DSM), which combines spatial modelling with distance sampling [41,48]. This allowed us to extrapolate to a larger area by combining data from sites with intensive sampling and random placement of transects (designed survey) with those from sites with few transects without random placement (undesigned survey) [31]. This is the first DSM for roe deer at this large scale of 70,000 km^2^ and a total sampling effort of 102.4 km of transects. In comparison, the study of Valente et al. [32] reported on a roe deer DSM with 635 km^2^ and 26 km transects. In addition, we provided a quantitative analysis of the quality of our extrapolation [36]. This method allowed us to combine field data with spatially-explicit covariates to determine environmental and management factors that significantly influenced the seasonal relative abundance of roe deer in a climate and land-use gradient. Consistent with previous studies [67,68], climate and land-use variables were predictive of roe deer relative abundance. In addition, the type of forest ownership, which can be a proxy for the management scheme, was also found to significantly influence the relative abundance.

Distance sampling of roe deer dung provides estimates of relative abundance from a snapshot of the period prior to the actual field survey, with its length depending on the decay rate of the dung [41]. Decay rates should be determined specifically for a given site; however, a previous study done in a forest in Poland gave a rough estimate of the possible season-specific decay rates. Roe deer dung deposited in late autumn and early winter persisted for an average of 118 days, in spring for 70 days and in summer for only an average of 26 days [69]. Only a few (17%) transects for the spring model were sampled in summer, and those detections accounted for only 4% of all detections in the spring model, this small inclusion of summer data may therefore have minimal influence on the relative abundance estimates. Based on the decay rate values from literature and the survey schedule, the results of the spring model are therefore an estimate of relative abundance primarily from winter to spring, while the autumn model represents the period from late summer to autumn. The differences in the significant variables for these two models provide insights on the changes in food availability and shelter between the two periods [70]. The spring model captures both the harsh conditions of winter and food-rich start of the growing season in spring and tentatively excludes dung placed by offspring which are born in May/June [71] due to differences in size and consistency (and possibly more difficult to detect) compared to adult dung. Therefore, we consider the spring model a pre-birth-pulse estimate of relative abundance. This sampling period also includes the start of the hunting season in Bavaria (1 May for bucks and female yearlings, [72]). 

Roe deer generally prefer heterogeneous habitats, such as early successional woodlands [73], but may forage in meadows and agricultural fields [3]. A higher proportion of forests and open habitats (arable, grasslands and shrublands) was beneficial for roe deer in the spring model. The affinity to woodlands in this model could be partially due to food availability in this season [31,74], but is more likely due to better cover to reduce risks posed by hunting [75], thermoregulation in winter [76] and shelter for offspring in Spring. The importance of meadows and arable land supports the findings of previous studies that food in these areas is of higher quality, especially in spring, and, therefore, is preferred by roe deer [77,78].

The autumn model rather reflects the summer conditions, when there is plenty of crop yield in arable fields, but also the constant threat from hunting, which is the main cause of roe deer mortality in the absence of predators [4]. In Bavaria, the hunting season for bucks is from the 1 May until the 15 October, for does and fawns between the 1 September and 15 January, for female yearlings between the 1 May and the 15 January [72]). This may explain the importance of edge proximity rather than individual habitat types on relative abundance estimates. Our results show that relative abundance is higher in areas close to a forest edge, where roe deer can easily commute between forests and fields [79]. Studies on the effects of hunting on the behavior of roe deer have shown that these ungulates adjust their behavior during the hunting season by being more vigilant [80] and using more woodlands during the day and open fields at night [75]. In contrast to the spring model, hunting was present during the entire period covered by the autumn model. This may explain why the relationship between relative abundance and the percentage of grasslands and shrublands in this open habitat, where animals are highly visible, changed from a positive relationship in the spring model to a negative relationship in the autumn model. The total pellet abundance was higher in the autumn model (post-birth-pulse model). A possible reason for this is the addition of spring-born fawns [81]. Furthermore, in both seasons, a higher proportion of private forests positively contributed to relative roe deer abundance, while the effect of state forests was not significant. Since there are no predators in most of Bavaria, with the exception of lynx (*Lynx lynx*) in the East, populations of ungulates are controlled by hunting [82]. Hunting quotas are commonly based on browsing pressure; however, actual hunting pressure may differ due to the different motivation of hunters in different forest types [83]. Hunters in most non-state forests tend to hunt rather recreationally and may be rather motivated by good hunting opportunities, while foresters of state forests argue that hunting should be driven by reducing ungulate densities to promote natural forest regeneration [82]. As such, differences in relative abundance between state-owned and non-state-owned forests could reflect differences in the motivation of hunters in the respective ownership types.

Climate affects roe deer populations both directly (e.g., harsh winters; [84,85]) and indirectly (e.g., trophic interactions; [86,87]). Our GAM results highlighted the importance of winter conditions on relative abundance. Of all seasonal variables, only mean temperature in winter was significant in our model, with intermediate values being favorable to the roe deer. Winter conditions have been shown to be limiting for roe deer due to higher thermoregulation and locomotion costs coupled with less food availability [24,88,89]. Seasonal temperature ranges, rather than mean temperature, were significant for all other seasons. Harvest density was also lowest in the areas with the coldest climate in agricultural and near-natural landscapes. Our models overestimated abundance as compared to the harvest data in the same areas; this may also be due to stronger seasonality and seasonal shifts in home ranges due to migration [90]. Unfortunately, in our models, predictions in these extreme climates for spring and autumn were often discarded in the extrapolation evaluation. Data was limited and insufficient for some areas, but the quantitative evaluation of the extrapolation allowed us to automatically identify these areas of uncertainty and exclude them from further analysis. The uncertainty of the predictions in the coldest areas arises from limited data. Our focal plots with intensive sampling and random arrangement of transects did not include sites in climate 1 due to limitations in resources. We therefore had very limited data for climate 1 and the covariate values were often outside the fitted range. We recommend extra sampling effort in environments at the endpoints of the climate continuum and emphasize the importance of the quantitative evaluation when extrapolating predictive models. 

At the landscape scale, our results showed that the highest abundance of roe deer is expected in agriculture-dominated landscapes and the lowest in urban landscapes for both seasons. It is well known that roe deer is well adapted to fragmented landscapes due to their foraging [2] and behavioral [91] plasticity. While historically roe deer occurred in predominantly forested environments, they have been taking advantage of a wide variety of habitats, including agricultural plains. Habitats providing a heterogeneous matrix of agriculture and shelter belts and small woodland structures may especially support high densities [92]. While being a very flexible and adaptable species, the lower abundance in urban landscapes might be attributed to the fact that less suitable habitat in terms of forage and shelter is available in these landscapes. Also, human settlements carry a higher risk of encounters with hunters and dogs, and also a higher risk of vehicle collisions [3,93,94]. 

The potential of DSM using an indirect method of distance sampling for population size estimations and ecological assessment has been previously recognized [32]. Advantages include the simplicity of the field survey, the ability to predict relative abundance in areas not covered by the survey, and the insights gained from the relationship between abundance and various covariates. The limitations of this first attempt to estimate relative abundance at a larger scale should serve as recommendation for future surveys and monitoring programs. The first challenge was the time-period covered by the field survey. Our spring survey slightly extended into summer; it was expected that the distribution of resources used by roe deer had changed within this period. However, since the majority of the transects and detections were in spring itself, the results are solid, but it would be better if the period for sampling was reduced. In autumn, we were able to prevent this limitation, and consequently, the autumn survey was restricted to a shorter period. The second challenge was that a considerable number of observers was needed for the field surveys across Bavaria. MCDS [47] would provide the means to account for the differences in detection rates per observer; however, we suggest that each observer should satisfy the minimum of 70–80 observations [48] to avoid pooling of observers and to obtain an independent detection function for each observer. 

We suggest that the relative abundance estimates as an index and the insights into the relationships with land cover types, climate and management variables in this study are of considerable value for data-based management in itself [23]. Yet, it must be noted that relative abundance could be converted into an absolute measure of population size by adding the defecation and decay rates of dung as an offset to the DSM [32]. Previous studies have used a common rate for the entire study area [11,15]; however, this should not be the case for larger scale studies, as production and decay rate depend on various factors such as deer habitat and local climate variables such as temperature and precipitation [95,96]. The ideal monitoring program for absolute population size should, therefore, include habitat-specific experiments to estimate production and decay rates. 

## 5. Conclusions

The findings of this study provide important insights for the management of roe deer in human-modified landscapes. Europe has the highest rate of human-induced forest fragmentation in the world [97]. Habitat fragmentation, which is characterized by habitat loss, has consistent negative impacts on biodiversity [98]. However, this may not apply to a generalist species such as the roe deer, which we have shown to be relatively more abundant in agricultural than in near-natural landscapes. This supports the hypothesis that deer may be able to exceed the carrying capacity of woodlands due to their adaptability to agricultural landscapes [99]. Especially in landscapes where agriculture provides extra nutrition, densities in adjacent forests may be too high to promote forest regeneration. In the absence of predators, hunting plays an important role in the management of this species. However, our results show that private forests are linked to higher relative abundances, which could also be due to differences in hunting pressure. There is a need for studies that focus on the importance of hunting for wildlife and forest management. 

Monitoring of abundance and distribution of large herbivores is challenging. The modelling methodology that we used allowed us to create abundance distribution maps that can be useful for resource managers and non-experts. Despite the limitation to relative abundances rather than absolute abundances, these estimates can still be useful for data-based management interventions. 

However, the challenge of validating model predictions remains. Agreement with harvest data from hunting statistics was not expected, as harvest data may be biased and does not account for effort [100]; however, we found a significant medium degree of correlation. Other possibilities for validation include data from direct surveys such as those using thermal infrared cameras and also with other indices of animal abundance, such as the vehicle collision index [68], browsing index [101] and estimates of occupancy from camera traps [102]. We focused on a single important species; however, future studies should explore modelling relative or absolute abundances of coexisting species and to relate this with long-term fitness data.

## Figures and Tables

**Table 1:** In total, 26 covariates were considered and included (✔) in the density surface models. A variable was removed if it was correlated with an ecologically more relevant variable (Pearson’s r > |0.7|), marked as x (1 correlated with winter mean temperature; 2 correlated with summer mean temperature; 3 correlated with arable; 4 correlated with summer temperature range).

Variable (Model Abbreviation)	Units	Data Source	Spring Model	Autumn Model
Coniferous forest (coniferous)	% cover	CORINE land cover 2018 [42]	✔	✔
Deciduous and mixed forest (deciduous_and_mixed) Arable land (arable)	% cover		✔	✔
Grass and shrublands (grass_and_shrubs)	% cover		✔	✔
Water and other unsuitable areas (water)	% cover		✔	✔
Artificial surfaces (artificial)	% cover		✔	✔
Winter mean temperature (win_mean_temp)	°C	German Meteorological Service (DWD) seasonal gridded data 2019 [52,53,54,55]	✔	-
Spring mean temperature (spr_mean_temp)	°C		x^1^	-
Summer mean temperature (sum_mean_temp)	°C		-	✔
Autumn mean temperature (aut_mean_temp)	°C		-	x^2^
Winter temperature range (win_temp_range)	°C		✔	-
Spring temperature range (spr_temp_range)	°C		✔	-
Summer temperature range (sum_temp_range)	°C		-	✔
Autumn temperature range (aut_temp_range)	°C		-	✔
Winter accumulated precipitation (win_acc_precip)	mm		x^1^	-
Spring accumulated precipitation (spr_acc_precip)	mm		✔	-
Summer accumulated precipitation (sum_acc_precip)	mm		-	✔
Autumn accumulated precipitation (aut_acc_precip)	mm		-	✔
Private forest (private_forest)	% cover	Forest overview map [56]	✔	✔
State forest	% cover		✔	✔
Corporate forest (corporate_forest)	% cover		✔	✔
NDVI seasonal mean (ndvi) *	-	MODIS/Terra Vegetation Index [51]	✔	x^3^
Elevation (elev)	degrees	EU-DEM v.1.0 [50]	x^1^	x^4^
Slope (slope)	degrees		✔	✔
Aspect (aspect)	m		✔	✔
Edge proximity (edge_proximity)	m	Proximity raster generated from distance to woodlands in CORINE 2018 [42]	✔	✔

* spring model—mean NDVI from the start of winter 2018 to the last day of sampling in summer; autumn model—mean NDVI from summer to autumn.

**Table 2:** Comparison of seasonal GAM models using tweedie and negative binomial distribution. The model outcome is the estimated relative abundance of roe deer dung pellet groups in each segment. Offset is the area of the segment. For variable names, see Table 1. The model formula shows the summation of the significant smooth (*s*) terms after model selection.

Season	Distribution	Model Formula	AIC (∆AIC)	% Deviance Explained
Spring	Tweedie	*s*(coniferous) + *s*(deciduous_and_mixed) + *s*(arable) + *s*(grass_and_shrubs) + *s*(private_forest) + *s*(win_mean_temp) + *s*(win_temp_range) + *s*(spr_acc_precip) + offset	1046.11 (0)	32.8%
Spring	Negative binomial	*s*(artificial) + *s*(win_mean_temp) + *s*(spr_acc_precip) + offset	1064.06 (17.95)	14.7%
Autumn	Tweedie	*s*(edge_proximity) + *s*(private_forest) + *s*(corporate_forest) + *s*(grass_and_shrubs) + *s*(sum_temp_range) + *s*(aut_temp_range) + offset	1177.12 (0)	34.3%
Autumn	Negative binomial	*s*(edge_proximity) + *s*(corporate_forest) + *s*(grass_and_shrubs) + *s*(sum_temp_range) + s(aut_temp_range) + offset	1248.35 (71.23)	21.9%

## Data Availability

Data are available upon request from the corresponding authors.

## Supplementary Materials

The following supporting information can be downloaded at: https://www.mdpi.com/article/10.3390/ani12030222/s1, Table S1: Land cover classification, Table S2: Comparison of detection functions, Figure S1: Comparison of models with tweedie and negative binomial distributions with quantile-quantile plots, Figure S2: Maps of Coefficient of variation (CV) associated with the abundance estimation, Figure S3: Comparison of relative abundance between landscape types and between seasons.
