# Supplementary material for: Modelling the Relative Abundance of Roe Deer (Capreolus capreolus L.) along a Climate and Land-Use Gradient
Source: Animals (Basel). 2022 Jan 18;12(3):222. doi: 10.3390/ani12030222 (PMC8833417; doi:10.3390/ani12030222)
Supplement: Supplementary file 1 [file animals-12-00222-s001.zip › animals-1535992-supplementary.pdf]

animals

MDPI

Table S1. Land cover classification based on CORINE 2018 [41]. Re-classification 1 was used for selection of study quadrants and plots, and for stratification of effort in the survey design. The percent cover of re-classification 2 was used as covariate in the density surface model. Unsuitable areas are those that are highly unlikely as roe deer habitat.

Code	Land cover class	Re-classification 1	Re-classification 2
111	Continuous urban fabric	Artificial surfaces	Artificial surfaces
112	Discontinuous urban fabric	Artificial surfaces	Artificial surfaces
121	Agricultural farms	Artificial surfaces	Artificial surfaces
122	Road and rail networks and associated land	Artificial surfaces	Artificial surfaces
123	Port area	Artificial surfaces	Artificial surfaces
124	Airports	Artificial surfaces	Artificial surfaces
131	Mineral extraction sites	Artificial surfaces	Artificial surfaces
132	Dump sites	Artificial surfaces	Artificial surfaces
133	Construction sites	Artificial surfaces	Artificial surfaces
141	Green urban areas	Artificial surfaces	Artificial surfaces
142	Sport and leisure facilities	Artificial surfaces	Artificial surfaces
211	Non-irrigated arable land	Agricultural areas	Arable
221	Vineyards	Agricultural areas	Arable
222	Fruit tree and berry plantations	Agricultural areas	Arable
231	Pasture, meadows and other permanent grasslands under agricultural use	Agricultural areas	Grass and shrublands
242	Complex cultivation patterns	Agricultural areas	Grass and shrublands
243	Land principally occupied by agriculture, with significant areas of natural vegetation	Agricultural areas	Grass and shrublands
311	Broad-leaved forest	Forest and semi-natural areas	Deciduous and Mixed forest
312	Coniferous forest	Forest and semi-natural areas	Coniferous forest
313	Mixed forest	Forest and semi-natural areas	Deciduous and Mixed forest
321	Natural grassland	Forest and semi-natural areas	Grass and shrublands
322	Moors and heathland	Forest and semi-natural areas	Grass and shrublands
324	Transitional Woodland / shrub	Forest and semi-natural areas	Grass and shrublands
331	Beaches, dunes and sand plains	Forest and semi-natural areas	Water and other unsuitable areas

332	Bare rock	Forest and semi-natural areas	Water and other unsuitable areas
333	Sparsely vegetated areas	Forest and semi-natural areas	Water and other unsuitable areas
334	Burnt areas	Forest and semi-natural areas	Water and other unsuitable areas
335	Glaciers and perpetual snow	Forest and semi-natural areas	Water and other unsuitable areas
411	Inland marshes	Wetlands	Water and other unsuitable areas
412	Peatbogs	Wetlands	Water and other unsuitable areas
421	Coastal Salt marshes	Wetlands	Water and other unsuitable areas
423	Intertidal flats	Wetlands	Water and other unsuitable areas
511	Water courses	Water bodies	Water and other unsuitable areas
512	Water bodies	Water bodies	Water and other unsuitable areas
521	Coastal lagoons	Water bodies	Water and other unsuitable areas
522	Estuaries	Water bodies	Water and other unsuitable areas
523	Sea and ocean	Water bodies	Water and other unsuitable areas

Table S2. Comparison of detection functions for the spring (top) and autumn (bottom) distance data.

Key	Adjustment	Formula	AIC (ΔAIC)	CvMises p-value
hn	Cosine	~1	367.20 (37.35)	0.54
	Hermite polynomial / Simple polynomial / null	~1	380.51 (50.66)	0.007
hr	Cosine / Hermite polynomial / Simple polynomial / null	~1	364.77 (34.92)	0.53

hn	null	~Bare ground / ~grass and forb / ~shrubs low in height / shrubs mid / shrubs high	Models failed to fit	
hn	null	~Observer group	331.46 (1.61)	0.086
hr	null	~Bare ground / ~grass and forb / ~shrubs low in height / shrubs mid / shrubs high	Models failed to fit	
hr	null	~Observer group	329.85 (0)	0.39
hn	Cosine	~1	19.72 (27.39)	0.35
hn	Hermite polynomial / Simple polynomial / null	~1	42.48 (50.15)	0.003
hr	Cosine	~1	18.04 (25.71)	0.35
hr	Hermite polynomial / Simple polynomial / null	~1	18.15 (25.82)	0.33
hn	null	~Bare ground / ~grass and forb / ~shrubs low in height / shrubs mid / shrubs high	Models failed to fit	
hn	null	~Observer group	15.07 (22.74)	0.007
hr	null	~Bare ground / ~grass and forb / ~shrubs low in height /shrubs mid / shrubs high	Models failed to fit	
hr	null	~Observer group	-7.67 (0)	0.49

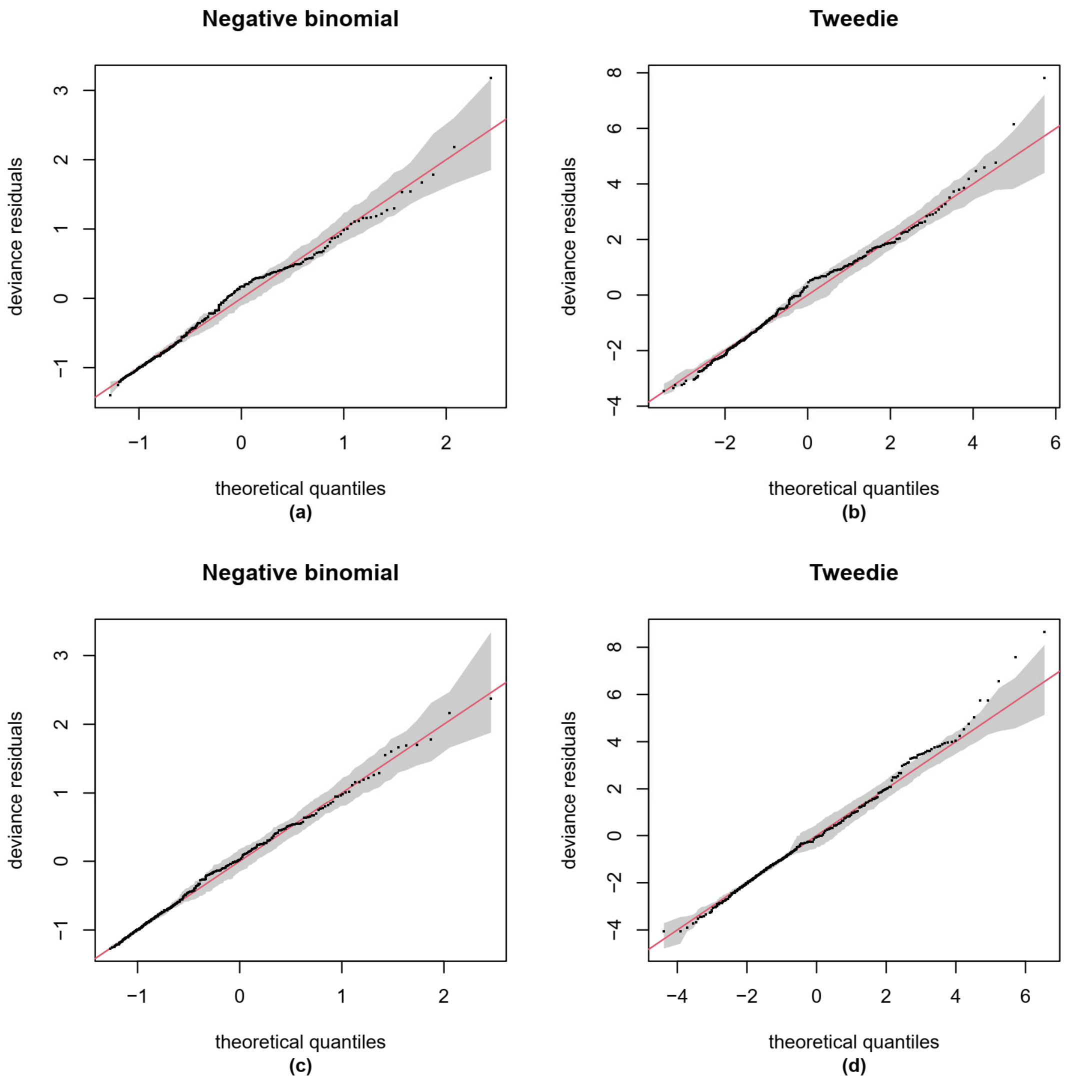

Figure S1. Comparison of models with tweedie and negative binomial distributions with quantile-quantile plots for spring (**a**,**b**) and autumn (**c**,**d**). Agreement between theoretical and observed (deviance residuals) indicates good model fit. Shaded regions are 90% reference bands. The tweedie points fall closer to the red line and this distribution was selected for the GAM models.

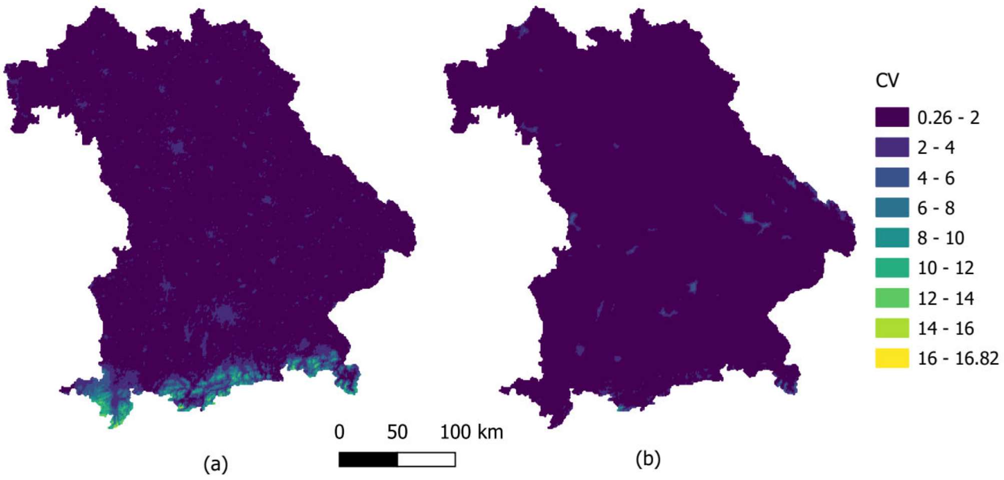

Figure S2. Maps of Coefficient of variation (CV) associated with the abundance estimation for the (**a**) spring model and (**b**) autumn model.

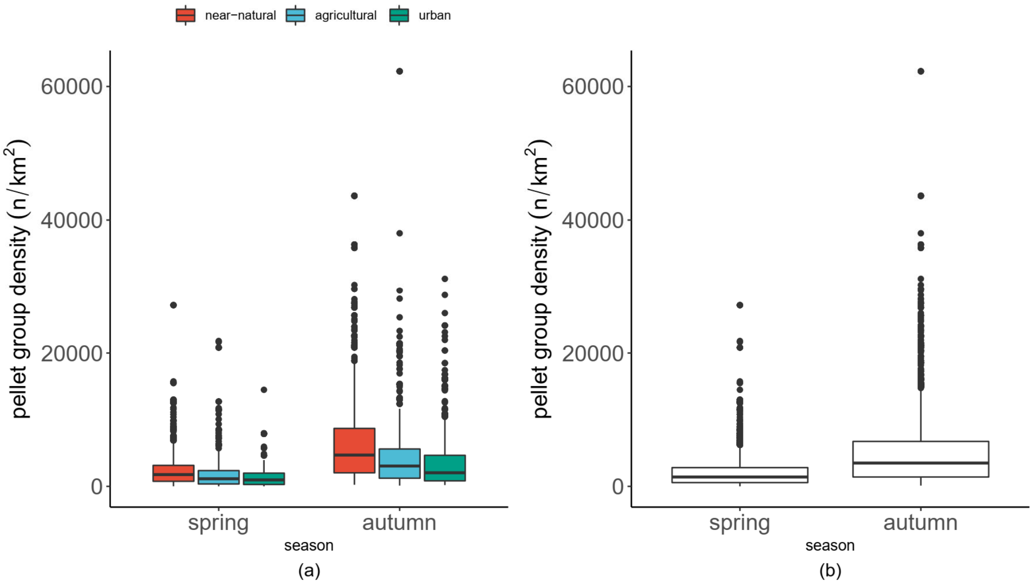

Figure S3. Comparison of relative abundance between landscape types and seasons. Relative abundance is presented using pellet group density (n/km^2).
